# Physicians' Characteristics Associated with Their Attitude to Family Presence during Adult Cardiopulmonary Resuscitation

**DOI:** 10.1155/2020/4634737

**Published:** 2020-10-20

**Authors:** Ali A. Al bshabshe, Mohammad Y. Al Atif, Mohammed A. Bahis, Abdulrahman M. Asiri, AbdulAziz M. Asseri, AbdulRahman A. Hummadi, Awad Al-omari, Yasser M. Almahdi, A. Rauoof Malik

**Affiliations:** ^1^Department of Medicine, College of Medicine, King Khalid University, P. O. Box 25216, Abha, Saudi Arabia; ^2^Department of Family Medicine, Armed Forces Hospital Southern Region, Khamis Mushayt, Saudi Arabia; ^3^Department of Critical Care, Aseer Central Hospital, Abha, Saudi Arabia; ^4^Department of medicine, King Faisal Medical City Southern Region, Abha, Saudi Arabia; ^5^Department of Medicine, Asser Central Hospital, Abha, Saudi Arabia; ^6^Department of Medicine, Samtah General Hospital, Jizan, Saudi Arabia; ^7^Department of Critical Care, Dr. Suliman AlHabib Medical Group, Riyadh, Saudi Arabia; ^8^Department of General Surgery, Armed Forces Hospital Southern Region, Khamis Mushait, Saudi Arabia; ^9^Department of Cardiology, Primacare Clinics, Avivo Group, Bur Dubai, Dubai, UAE

## Abstract

Healthcare providers have disparate views of family presence during cardiopulmonary resuscitation; however, the attitudes of physicians have not been investigated systematically. This study investigates the patterns and determinants of physicians' attitudes to FP during cardiopulmonary resuscitation in Saudi Arabia. A cross-sectional design was applied, where a sample of 1000 physicians was surveyed using a structured questionnaire. The study was conducted in the southern region of Saudi Arabia for over 11 months (February 2014–December 2014). The collected data was analyzed using the Pearson chi-square test. Spearman's correlation analysis and chi-square test of independence were used for the analysis of physicians' characteristics with their willingness to allow FP. 80% of physicians opposed FP during cardiopulmonary resuscitation. The majority of them believed that FP could lead to decreased bedside space, staff distraction, performance anxiety, interference with patient care, and breach of privacy. They also highlight FP to result in difficulty concerning stopping a futile cardiopulmonary resuscitation, psychological trauma to family members, professional stress among staff, and malpractice litigations. 77.9% mostly disagreed that FP could be useful in allaying family anxiety about the condition of the patient or removing their doubts about the care provided, improving family support and participation in patient care, or enhancing staff professionalism. Various concerns exist for FP during adult cardiopulmonary resuscitation, which must be catered when planning for FP execution.

## 1. Introduction

In clinical medicine, allowing family members to be present at a patient's bedside during cardiopulmonary resuscitation (CPR) is a controversial issue [1]. There has been growing support for family presence (FP) during CPR with medical practice becoming increasingly nonpaternalistic [2–4]. It allows a family member to afford visual or physical contact with the patient's undergoing cardiopulmonary resuscitation. Most studies confirm that it satisfies the patients' need to be with their loved ones in a critical illness [2–5]. Evidence from the literature suggests that FP at resuscitation enables family members to communicate information to the health team and provides spiritual and emotional support to the patients [2–4].

Some of the previous studies have shown that families like to be involved in the final moments to allow for more dignified closure and some religious or cultural rights [5, 6]. Twibell et al. [7] documented that FP contributes in developing a confident and peaceful environment that alleviates decisional burdens on patients, provides support for families, and reduces anxiety of the health care teams. Studies reveal that FP practice allows healthcare professionals (HCP) to perceive the patients, not as a disease but an individual who belongs to someone [2–4]. This improves care quality and enhances satisfaction with healthcare services [5]. Australian Resuscitation Council [8], European Resuscitation Council [9], American Heart Association [10], and the public have supported the FP during CPR [2]; however, this remains a divided area among the HCPs (Health Care Professionals) with support ranging from 3 to 98 percent [11]. Despite the immense support, anecdotal studies have regarded it as a controversial issue, given the diverging findings of the researches and practitioners [12–14]. Objectively, several professional bodies have published recommendations or expressed opinions in favor of FP during CPR suggesting no adverse effects, and some possible benefits of FP during CPR [15–17].

The practice of FP during CPR was just restricted to Western countries; however, recently healthcare professionals of the non-Western countries became aware of the significance of this practice. Both positive and negative opinions have been observed from health-care staff and family members with regard to this practice. The major obstacle in the acceptance of FP is HCP's fear regarding its negative consequences. Moreover, the current clinical practice brings about contrasting views from both the groups and tends to create conflict between HCPs and family members. However, this remains an unexplored area for Saudi Arabia, where the attitude and characteristics of HCPs have not been explored. One of the previous researches conducted in Saudi Arabia was central to perception of the HCP [18], while other studies have been restricted to regions such as Poland [9], Brazil [19], Hong Kong [20], Australia [11], and UK [21]. The study aims to bridge this gap by examining the physicians' attitudes toward FP during adult CPR in Saudi Arabia.

Previously, the attitudes of only nonphysician HCPs were considered for FP during CPR. However, few of the previous studies considering physicians showed that the majority of them opposed FP during CPR [22–24]. Therefore, there is a need to focus on the attitudes of HCPs about CPR to identify the predictors that hinder or facilitate this practice. Also, to the best of the researcher's knowledge, the attitudes of physicians working in the Middle Eastern countries to FP during CPR have not been studied systematically. Therefore, the present study has investigated physicians' attitudes toward FP in Saudi Arabia.

## 2. Materials and Methods

### 2.1. Study Design

The study has employed a cross-sectional design to collect data related to the physician's attitudes towards family presence during resuscitation in Saudi hospitals. The time duration between February 2014 and December 2014 has been focused. This study was undertaken using a questionnaire that was distributed among physicians in different hospitals.

### 2.2. Study Participants

**Figure 1:**
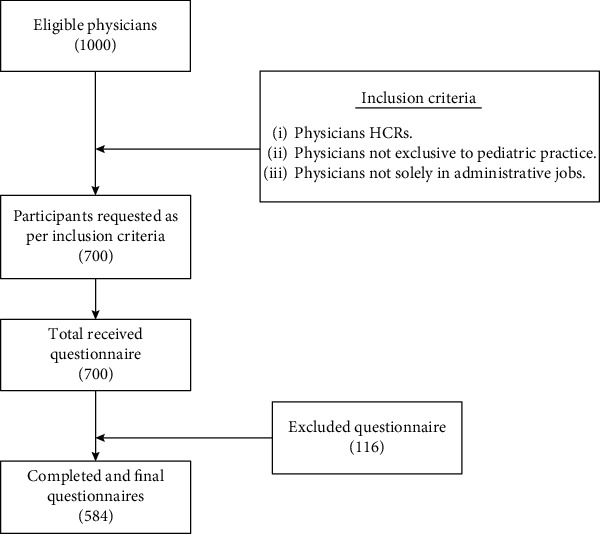
Study procedure.

The study participants were sampled using database of different hospitals, and the physicians were contacted through email. This database included members of the national council within the hospitals. The inclusion criteria for physicians were those who were working in the clinical setting with not less than 1 year of working experience (Figure 1). While physicians with <1-year experience were excluded from the study. The overall sample included 1000 physicians; however, only 700 agreed to fill in the questionnaire. The questionnaire was only sent to the physicians, who agreed to participate in the study. The sample included interns, resident physicians, consultants, and specialists. The reason for including interns was to know their opinion about FP during CPR, as they are in their learning phase. The received questionnaires were screened for compliance. Out of 700 questionnaires, only 584 were returned with complete information (response rate of 83.4%). The reason for this high response rate was long study duration, i.e., 11 months, along with providing self-selection survey mode and sending multiple reminders to the physicians.

### 2.3. Ethics Approval

The study was approved by the Institutional Review Board of King Khalid University, Abha. Each physician signed the consent form, and all the physicians were assured of the confidential and anonymous handling of the data. The Research Ethics Committee approved the study at King Khalid University College of Medicine, reference number (REC# 2014-02-12).

### 2.4. Data Collection

The self-administered questionnaire was derived on different theories, including the health belief model [25], programmed behavior [26], the theory of reasoned action [27], and the theory of self-efficacy [28]. As no theory is employed in this research, therefore, the aspects discussed in each theory are combined to formulate the questionnaire. The validity of the behavioral theories for the study questionnaire has been established in the previous studies [29]. To test the validity of the questionnaire, a content validity test was carried out to ensure the appropriateness of the scale before data collection, as the instrument was tested for validity and reliability. The result of content validity index (CVI) was 0.89 that indicates relevancy of the items to the research objectives.

The questionnaire was divided into two parts. The first part included questions related to the demographic information. The second part of the questionnaire included questions about whether the healthcare staff believed the practice of FP during CPR was beneficial to the family members. The self-efficacy to adequately handle such difficult situations, along with the influence of social pressure in hindering the practice, was also considered. The physicians' agreement and disagreement to the given practice was also focused. The responses to questions were graded on a five-point Likert scale ranging between 1 = strongly agree and 5 = strongly disagree.

Initially, the physicians were asked: “Do you agree that family members should be given the option to witness CPR on their relatives?” following their opinions regarding any potential “immediate” harmful (6 questions) or beneficial (6 questions) consequences of FP. Also, physicians were asked to give opinions regarding any risk of adverse psychological effects on family members, professional stress, and medical litigation. Lastly, two questions were asked, in which the physician was asked whether he/she would allow FP during CPR. And whether his clinical practice is impacted by FP.

### 2.5. Data Analysis

IBM SPSS (Statistical Package for Social Sciences) version 23.0 was used for the analysis. The variables were presented as mean ± SD or number (percent), while statistical significance was analyzed using the Pearson chi-square test. The association of physician characteristics with their responses was investigated using Spearman's correlation analysis (age) and chi-square test of independence (other physician characteristics). A two-sided *P* value of <0.05 was taken as the criterion of statistical significance.

## 3. Results

After excluding irrelevant and incomplete responses, 584 (82.1% male and 17.8% female) were considered eligible for analysis (Table 1). The table shows that 35.6% surgeons performed CPR as it is an important part of some surgical procedures, followed by internist (27.1%), others (26.0%), ER physician (8.6%), and ICU physician (8.0%). They agreed that discontinuing futile CPR might be difficult in the presence of family members.

Concerning the immediate undesirable effect, most of the physicians agreed that FP increase the staff performance anxiety (84.9%), followed by privacy breach (76.9%) with increased possibility of family interference (70.0%) (Table 2).

Table 3 presents the views of physicians about potential immediate desirable effects of FP during CPR. Very few physicians agreed that FP could enhance family support towards staff (10.3%), improve family participation (7.4%) in subsequent care of the patient, or increase staff professionalism (9.9%).

Table 4 presents the views of physicians about potential delayed effects of FP during CPR. About 90% of physicians believed that FP may increase professional stress among staff, and about half of them thought FP would increase the chances of malpractice litigation.

**Figure 2:**
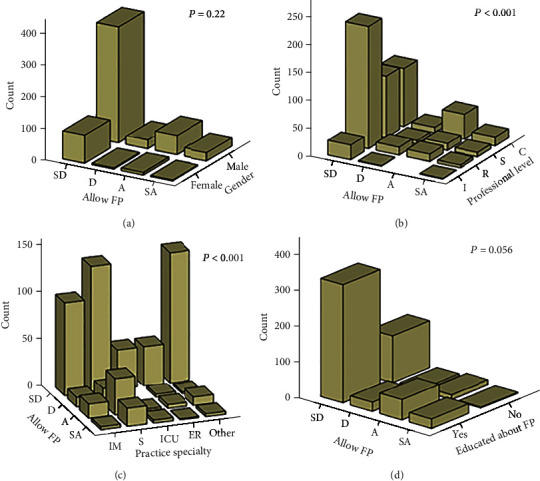
Association of physician characteristics with their opinions on potential effects of family presence during cardiopulmonary resuscitation. (a) Gender. (b) Professional level. (c) Practice specialty. (d) Education level. ^∗∗^(SA: strongly agree; A: agree; D: disagree; and SD: strongly disagree) (C: consultant; S: specialist; R: resident; and I: Intern) (IM: internal medicine and related; S: surgery and related; ICU: intensive care unit; ER: emergency room; or others).

There was a poor association between physicians' characteristics and their willingness to allow FP during CPR (Figure 2). The findings showed that most of the physicians were against allowing FP during CPR, based on the physicians' gender, their professional level, practice specialty, and education about FP.

Table 5 shows the results for the last two questions, which reported that physicians would not apply FP during CPR (80%), while 68% responded that FP impacts their CPR practices.

## 4. Discussion

In this cross-sectional study, the majority of the physicians opposed FP during CPR. The physicians were concerned that FP might decrease bedside space available for the CPR team, produce staff distraction and performance anxiety, interfere with patient care, jeopardize privacy, and make the decision to discontinue futile CPR difficult. The majority of the physicians included in this study stated that FP during CPR could increase the risk of adverse psychological reactions among family members, professional stress among staff, and the temptation to open up malpractice suits. However, one of the studies contradicted these findings stating that family members witnessing CPR were able to tolerate the situation well and found acceptance and adjustment to their loss easier without any risk of psychological morbidity [15, 16, 30, 31].

The findings of the present study perceived a number of negative consequences such as anxiety among family members and promotion of their cooperation with staff, which are similar to those expressed in previous studies [32, 33]. However, a significant amount of evidence from existing literature contradicts these concerns and supports the positive effects of FP [34–36]. These findings emphasize that physicians believed that FP affects the safety of resuscitation, such as the family may indirectly or directly increase staff stress, impact coordination, which increases the possibility of medical errors; however, it does not mean that is actually the case. Just because physicians in this study had concerns about FP during CPR does not mean it is a bad idea, as there are many organizations favoring this practice.

The present study depicted that educating family about patient's condition has a significant impact on the attitudes of HCPs towards FP during CPR. This is corroborated by earlier studies that endorse a positive attitude of senior-level physicians, and physicians with prior knowledge on FP during CPR [35–37]. The physicians recruited in the present study were in favor of having defined institutional policies for FP during CPR that validate the propositions generated by some earlier investigators. There are several hospitals demonstrating the positive impact of administrative guidelines [5, 38, 39]. The difference in the findings may also be attributed to the different regional, cultural, and religious factors [36]. American College of Chest Physicians [23] and online polling survey [34] also concluded similar results and showed that FP is not supported during CPR by the majority of the physicians. On the contrary, a study conducted by Leung and Chow [40] showed that the majority of the physicians supported FP during CPR, if family members could share the dying moments with patients. However, these physicians refrain from FP only if family presence will increase the risk of litigation and colleagues during CPR. There is need to enhance the acceptance of this practice and making it the universal standard of care through training and education programs to establish appropriate administrative policies [40].

The findings of the study recommend that the practice of FP in CPR need to follow a certain procedure to improve HCPs' role as facilitator. Society of Critical Care Medicine (SCCM) family-centered care guidelines can be implemented for executing FP during CPR [41]. The guidelines explain that firstly it is important to identify whether family members are present and, if so, then the information of the resuscitation (ongoing) is communicated to them. Secondly, it is also important to determine whether FP is appropriate or not based on their emotional state (i.e., their willingness to be in the resuscitation). Family members should be communicated about what to expect and where to stand, with restricted number of family members. Additionally, HCPs should provide emotional support, and the efforts being instigated. Implementation of these recommendations would support FP during CPR.

## 5. Conclusions

The present study has investigated the patterns and determinants of physicians' attitudes to FP during cardiopulmonary resuscitation. It is believed that high education level leads to more acceptance of FP. The view of family members holds significant importance as to whether they really want to be present during resuscitation for both adults and pediatric patients in Saudi Arabia. Basically, the physicians feel threatened by the change of the unpredictable nature of resuscitation events observed by a family member having an emotional involvement with the patient. Conversely, evidence suggests that FP during CPR can have positive effects such as acceptance and adjustment to their loss easier without any risk of psychological morbidity. Consequently, many hospitals across the world are now promoting the practice. Although the study helps to understand the barriers to the general acceptance of FP during CPR, larger multicenter studies are needed to clearly define the magnitude and impact of regional variations in the attitude of physicians to this important practice issue in clinical medicine. There is also need to further study other health care providers' attitudes regarding family presence during resuscitation.

## Figures and Tables

**Table 1:** Characteristics of the responding physicians.

Characteristic	Mean ± SD or number	(Minimum, maximum) or (%)
Age, years	35.61 ± 9.58	(21, 64)
Gender		
Male, *n* (%)	480	(82.1%)
Female	104	(17.8%)
Professional level		
Consultant, *n* (%)	172	(29.5%)
Specialist, *n* (%)	135	(23.1%)
Resident, *n* (%)	249	(42.6%)
Intern, *n* (%)	28	(4.8%)
Specialty		
Internist, *n* (%)	127	(21.7%)
Surgeon, *n* (%)	208	(35.6%)
ICU physician, *n* (%)	47	(8.0%)
ER physician, *n* (%)	50	(8.6%)
Others, *n* (%)	152	(26.0%)
Educated about the subject	151	(25.9%)

ICU: intensive care unit; ER: emergency room. ^a^The category “others” included 81 ears, nose, and throat physicians; 49 anesthesiologists; 15 family physicians; and 7 gynecologists.

**Table 2:** Physicians' view about potential immediate undesirable effects of FP during CPR.

Assumed effect due to family presence (undesirable effects)	Physician response				*P*
	Strongly agree *N* (%)	Agree *N* (%)	Disagree *N* (%)	Strongly disagree *N* (%)	
Decreased bedside space for staff	277 (47.4%)	307 (52.6%)	0	0	<0.001
Staff distraction	61 (10.4%)	407 (69.7%)	109 (18.7%)	7 (1.2%)	<0.001
Staff performance anxiety	54 (9.2%)	496 (84.9%)	34 (5.8%)	0	<0.001
Interference by family members with patient care	140 (24.0%)	409 (70.0%)	35 (6.0%)	0	<0.001
Breach of privacy	134 (22.9%)	449 (76.9%)	1 (0.2%)	0	<0.001
Difficulty discontinuing failed CPR	50 (8.6%)	280 (47.9%)	254 (43.5%)	0	<0.001

**Table 3:** Physicians' view about potential immediate desirable effects of FP during CPR.

Assumed effect due to family presence (desirable effects)	Physician response				*P* value
	Strongly agree *N* (%)	Agree *N* (%)	Disagree *N* (%)	Strongly disagree *N* (%)	
Help educate family about patient's condition	2 (0.3%)	200 (34.2%)	382 (65.4%)	0	<0.001
Reduce family anxiety and fear	27 (4.6%)	48 (8.2%)	284 (48.6%)	225 (38.5%)	<0.001
Remove family doubts about care	40 (6.8%)	63 (10.8%)	426 (72.9%)	55 (9.4%)	<0.001
Improve family support to the staff	0	60 (10.3%)	299 (51.2%)	225 (38.5%)	<0.001
Improve family participation in patient care	0	43 (7.4%)	432 (74.0%)	109 (18.7%)	<0.001
Increase staff professionalism	58 (9.9%)	71 (12.2%)	254 (43.5%)	201 (34.4%)	<0.001

CPR: cardiopulmonary resuscitation. *P* values derived from Pearson chi-square using “equiprobability model”.

**Table 4:** Physicians' view about potential delayed effects of FP during CPR.

Assumed effect due to family presence	Physician response			*P*
	Increased risk *N* (%)	No effect *N* (%)	Decreased risk *N* (%)	
Psychological trauma to family members	184 (31.5%)	152 (26.0%)	248 (42.5%)	<0.001
Professional stress among the staff	529 (90.6%)	34 (5.8%)	21 (3.6%)	<0.001
Malpractice litigation against the staff	291 (49.8%)	236 (40.4%)	57 (9.8%)	<0.001

*P* values derived from Pearson chi-square using “equiprobability model”.

**Table 5:** Use of family presence (FP) during CPR.

Questions	Yes (%)	No (%)
Would you allow FP during CPR?	20%	80%
Does FP impact your clinical practice?	68%	32%

## Data Availability

The datasets used and analyzed during the current study are available from the corresponding author on reasonable request.
